# Functional superhydrophobic surfaces made of Janus micropillars

**DOI:** 10.1039/c4sm02216e

**Published:** 2014-11-21

**Authors:** Lena Mammen, Karina Bley, Periklis Papadopoulos, Frank Schellenberger, Noemí Encinas, Hans-Jürgen Butt, Clemens K. Weiss, Doris Vollmer

**Affiliations:** a Max Planck Institute for Polymer Research , Ackermannweg 10 , D-55128 , Mainz , Germany . Email: vollmerd@mpip-mainz.mpg.de; b University of Applied Sciences Bingen , Berlinstrasse 109 , D-55411 Bingen , Germany

## Abstract

We demonstrate the fabrication of superhydrophobic surfaces consisting of micropillars with hydrophobic sidewalls and hydrophilic tops, referred to as Janus micropillars. Therefore we first coat a micropillar array with a mono- or bilayer of polymeric particles, and merge the particles together to shield the top faces while hydrophobizing the walls. After removing the polymer film, the top faces of the micropillar arrays can be selectively chemically functionalised with hydrophilic groups. The Janus arrays remain superhydrophobic even after functionalisation as verified by laser scanning confocal microscopy. The robustness of the superhydrophobic behaviour proves that the stability of the entrapped air cushion is determined by the forces acting at the rim of the micropillars. This insight should stimulate a new way of designing super liquid-repellent surfaces with tunable liquid adhesion. In particular, combining superhydrophobicity with the functionalisation of the top faces of the protrusions with hydrophilic groups may have exciting new applications, including high-density microarrays for high-throughput screening of bioactive molecules, cells, or enzymes or efficient water condensation. However, so far chemical attachment of hydrophilic molecules has been accompanied with complete wetting of the surface underneath. The fabrication of superhydrophobic surfaces where the top faces of the protrusions can be selectively chemically post-functionalised with hydrophilic molecules, while retaining their superhydrophobic properties, is both promising and challenging.

## Abstract 2

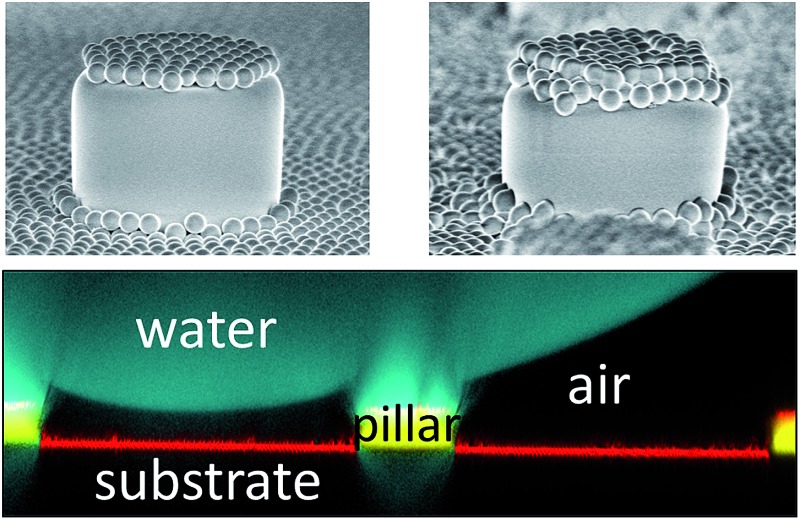
Particle coated micropillar arrays having hydrophobic sidewalls and hydrophilic silica tops are fabricated, enabling the top sides to be selectively post-functionalized. The so termed Janus pillars remain in the Cassie state even after chemical modification of the top faces.

Controlling the wetting^1^ of solid surfaces is of great interest in many fields, including microfluidics,^2–4^ spray painting and coating,^5,6^ fog harvesting,^7^ textile industry,^8^ and the deposition of pesticides on plant leaves.^9^ A step towards this goal has been the fabrication of superhydrophobic, superhydrophilic, and hydrophilic–superhydrophobic patterned surfaces.^9–12^ Superhydrophilicity can be achieved by a material with a rough surface topography and high surface energy.^13^ Decreasing the surface energy can render the surface superhydrophobic. Superhydrophobicity is defined by an apparent advancing contact angle of water with the surface above 150° and a roll-off angle below 10° (ref. 14 and 15) for drops of approximately 10 μL volume. Water drops roll off with little friction. This low adhesion is caused by air trapped between the drop and the substrate. The superhydrophobic state must be distinguished from the “Wenzel state”, in which the substrate is fully wetted by the liquid.^16^

Arrays of hydrophobic micropillars are models for superhydrophobic surfaces.^15,17–20^ A drop of water placed on such an array is only in contact with the top faces of the micropillars. The equilibrium apparent contact angle, *θ*_app_, of water on such surfaces has been calculated by minimizing the free energy of a drop assuming that the drop is in its global thermodynamic equilibrium. This assumption results in the Cassie–Baxter equation:^21^ cos *θ*_app_ = *f*(cos *θ* + 1) – 1, where *f* is the fraction of the solid surface in contact with water and *θ* is the Young's contact angle on a flat surface of the same material. The Cassie–Baxter equation leads to the requirement of a low-energy surface, *i.e.* a hydrophobic surface. Therefore, it was unclear whether a selective chemical post-functionalization of the top faces of superhydrophobic surfaces with hydrophilic molecules would be possible. Thus, the fabrication of superhydrophobic surfaces where the top face of each protrusion can be selectively chemically post-functionalised with hydrophilic molecules while retaining their superhydrophobic properties is both promising and challenging and has not been achieved yet.

Few strategies have been reported for creating hydrophilic spots with typical diameters of a few hundred microns on an otherwise superhydrophobic surface.^11,22^ These methods include microcontact or inkjet printing,^23^ photomasking,^24^ top-down lithography,^25^ and polymer deposition from solution.^26^ Water drops are confined to these hydrophilic spots while also wetting the underlying substrate. Furthermore, these post treatments are often harsh (UV), produce large pattern sizes (photomasking or printing), or are accompanied by the dissolution of the hydrophilic molecules used for functionalisation (*e.g.*, lipids and polymers) into the drop under investigation.^27^ Varanasi fabricated micropatterned surfaces *via* microcontact printing using a polydimethylsiloxane stamp.^28^ Part of the top faces can also be hydrophilized by evaporation or by pulling a drop over the surface. Depinning is accompanied by leaving tiny drops behind. If its mother drop contains non-volatile components these can alter the surface properties locally.^29^

Here, we introduce a method for the fabrication of transparent superhydrophobic micropillars with fluorinated hydrophobic sidewalls and functional hydrophilic silica tops, *i.e.*, Janus micropillars. We functionalised the top of each micropillar by chemically binding molecules of different hydrophilicities. The micropillar arrays were highly transparent, which enabled us to use laser scanning confocal microscopy to verify the existence of air cushions that separated the substrate from water. We demonstrate that superhydrophobicity in the arrays of Janus micropillars is maintained and show that the stability of the air cushions is determined solely by the properties of the hydrophobic rim of each micropillar, not by the hydrophilicity or chemical nature of the top faces of the micropillars.

## Methods

### Fabrication of silica-coated SU-8 micropillars

The flat-top cylindrical micropillars were fabricated by photolithography using a SU-8 photoresist (SI methods) and arranged on a glass slide in a square lattice.^30^ The micropillars were fluorescently labelled by first mixing the photoresist with a hydrophobic *N*-(2,6-diisopropylphenyl)-3,4-perylenedicarboxylic acid monoimide (PMI) dye^31^ at a concentration of 0.05 mg mL^–1^. The substrates were coated with silica by treatment with an O_2_ plasma for 30 s (at an O_2_ flow rate of 7 sccm), followed by immersion in a solution of tetraethoxysilane (1.82 mL) and ammonium hydroxide (28% in water, 4.2 mL) in ethanol (50 mL) for 2–3 h. Afterward, the substrates were rinsed with ethanol and dried in a N_2_ stream. Because SU-8 swells slightly in organic solvents, such as tetrahydrofuran, we observed some cracks in the silica shell after the washing step for PS removal (see below). These defects could be prevented by exposing the substrates to an O_2_ plasma (at an O_2_ flow rate of 7 sccm) for 1 h before decoration with the particles. The plasma penetrated the silica shell and removed an outer layer of SU-8, creating free space for swelling.

### Monolayer crystallisation procedure

The PS particles were synthesised by the soap-free emulsion polymerisation of styrene.^32^ The average diameter of the spherical and almost monodisperse particles was 1.1 μm or 1.4 μm (SEM). Highly ordered particle monolayers were prepared by self-assembly at the air–water interface of a Langmuir trough (242 cm^2^) using Milli-Q water (with a resistivity of 18.2 MΩ cm) as a subphase. Prior to use, the micropillar substrates were exposed to an Ar plasma for 4 min (at an Ar flow rate of 5 sccm) to remove any adhering organic impurities and stored in ethanol. The substrates were immersed into the subphase and placed on a holder parallel to the air–water interface. The particle dispersion (1.5 wt% in ethanol) was added dropwise *via* a tilted glass slide that was partially immersed in the subphase. After 15 min, the monolayer was compressed at a speed of 2 cm min^–1^ until a compact monolayer formed. This result manifested as an increase of the simultaneously recorded pressure. Thereafter, the particles were deposited on the substrates by lowering the water level, *i.e.*, a “surface-lowering transfer”. The particle micropillar arrays were fabricated by exposing the particle-decorated micropillars to an O_2_ plasma for 30 s (at an O_2_ flow rate of 7 sccm) and then coated with a thin silica shell as previously described.

### Fabrication of Janus (particle) micropillars

To form a film of PS particles, the particle-decorated micropillar arrays were exposed to toluene vapour for 1 h (to form Janus micropillars) or 40 min (to form Janus particle micropillars). The substrates were placed in a desiccator containing a vessel (with a 5 cm opening) filled with toluene. The substrates were then placed in a vacuum chamber to remove any solvent residues. After the sidewalls were hydrophobised (see below), the PS film was removed by thorough washing with THF, dichloromethane, methanol, ethanol, and Milli-Q water.

### Hydrophobisation

The micropillar arrays were hydrophobised using the chemical vapour deposition of 1*H*,1*H*,2*H*,2*H*-perfluorooctyl-dimethylchlorosilane.^33^

### SPPS protocol for GALA synthesis

Fmoc-Lys(Mca)-OH, Fmoc-Lys-(Dnp)-OH, all Fmoc-protected l-amino acids and preloaded resin (Fmoc-Gly-Wang resin, 100–200 mesh, loaded with 0.30 mmol g^–1^ of Gly) for SPPS were purchased by Novabiochem (Merck). The purity of the commercial amino acids was >98%. *N*-[(1*H*-Benzotriazol-1-yl)(dimethylamino)methylene]-*N*-methyl-methanaminium hexa-fluoro-phosphate *N*-oxide (HBTU, Novabiochem), ethyl cyanoglyoxylate-2-oxime (Oxyma Pure, Merck, >98%), *N*,*N*-iisopropylethylamine (DIEA, Fluka, >98%), trifluoroacetic acid (TFA, Acros, 99%), triisopropylsilane (TIS, Alfa Aesar, 99%), *N*-methyl-2-pyrrolidone (NMP, BDH, 99%), piperazine (Merck, >99%), fluorescein-5(6)-isothiocyanate (Sigma-Aldrich, >90%) and all solvents were used as received.

The peptide sequences were prepared using standard solid-phase Fmoc chemistry with a microwave assisted automated peptide-synthesizer (Liberty, CEM). The parameters used for coupling and deprotection steps are mentioned below and relate to 0.1 mmol of peptide. Coupling was achieved under 300 s of microwave heating, with the temperature reaching and stabilizing at 75 °C after around 90 s, with Oxyma Pure as an activator (5 equivalents), DIEA as a base (10 equivalents) and amino acid (5 equivalents). Then a first deprotection stage of 30 s (temperature reaching around 50 °C at the end) followed by a second cycle for 180 s (temperature 75 °C) with a 20 wt% solution of piperazine in DMF 3 was applied to remove the Fmoc protection group. The resin was washed 3 to 5 times between each coupling or deprotection step. The cleavage of the peptide from the resin was performed using a mixture of TFA/TIS/H_2_O (95%/2.5%/2.5%) for 15 h at ambient temperature. After filtration, the peptides were precipitated and centrifuged three times in cold diethyl ether, and dried in a vacuum.

### Functionalisation with FITC or GALA

The silica tops of the Janus micropillar arrays were amino-functionalised by dipping the substrate into a solution of aminopropyl-triethoxysilane (46 μL) in dry toluene (20 mL) for 1 h. The substrate was then rinsed with fresh toluene, dichloromethane, and ethanol. The substrate was functionalised with FITC by immersion into a solution of FITC (39 mg) in acetone (10 mL) for 1.5 h, followed by thorough rinsing with fresh acetone, dichloromethane, and ethanol.^34^ The silica tops were functionalised with the fluorescently labelled GALA peptide by being dipped overnight into a solution of dibenzylcyclooctyne-*N*-hydroxysuccinimide ester (DBCO-NHS ester, 0.1 mg L^–1^) in dimethylsulphoxide (DMSO, 5 mL), followed by washing with fresh DMSO and Milli-Q water (SI Methods). The DBCO-modified substrate was then immersed overnight in a solution of azide-functionalised GALA (0.1 mg L^–1^) in DMSO (5 mL), followed by thorough rinsing with fresh DMSO and Milli-Q water. The fluorescently labelled GALA was synthesised using standard Fmoc SPPS protocols using a CEM Liberty microwave-assisted solid phase peptide synthesiser. FITC was introduced at the N-terminus of the peptide by Fmoc-Lys(FITC)-OH, which was synthesised following a protocol by Fuchs *et al.* ^35^ All of the other Fmoc-amino acids, including Fmoc-Lys(N_3_)–OH, were commercially available. The identity of the peptide was confirmed using ^1^H-NMR (Bruker Avance 300), HPLC (Hewlett-Packard, Agilent), and MALDI-TOF-MS (Bruker-Daltonics).

### Instruments and characterization

The pillar arrays and particles were characterized by scanning electron microscopy (SEM) using a LEO 1530 Gemini instrument (Zeiss, Oberkochen, Germany) at low operating voltages (0.7–2 kV). The pillar arrays and their contact angles with water were imaged by inverted laser scanning confocal microscopy (LSCM, Leica, TCS SP5 II – STED CW) by applying glass substrates with a thickness of 170 μm. The LSCM has an absolute horizontal resolution of about 250 nm and a vertical resolution of about 1 μm. The spectral ranges could be freely varied, allowing the measurement of the emission from different dyes and the reflected light from the interfaces simultaneously. Water was labelled fluorescently with Alexa Fluor 488 at a concentration of 0.1 mg mL^–1^. The dyes (PMI and Alexa Fluor 488) were excited using the argon line at 488 nm. For treating the surfaces with argon or oxygen plasma a FEMTO plasma cleaner was used (Diener electronic, power: 15 W). Contact angle measurements were performed with a contact angle meter (DataPhysics; OCA35). Static contact angles were measured by depositing a liquid drop of 4 μL on the surface. Advancing contact angles were measured using a sessile drop of 4 μL, with a needle in it, and subsequently increasing the liquid volume at a rate of 0.5 μL s^–1^. Roll-off and receding angles were measured by depositing a drop of 5 μL and tilting the substrate at a rate of 2° s^–1^. They were determined in the moment when the drop detaches from the first outermost pillar.

### Surface area fraction in the nano-Cassie state

**Fig. 1:**
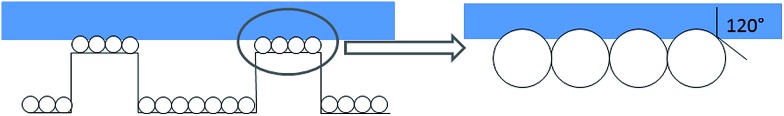
Sketch of micropillars decorated with particles.

The structure is made of a square array of cylindrical pillars with diameter *d*, height *h* and pitch *P* (Fig. 1). The particles have radius *r* and form a close-packed monolayer. Each particle occupies an area in the horizontal plane of ≈
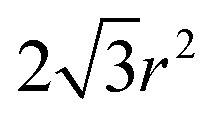
. Thus the number of particles on top of one pillar is about 
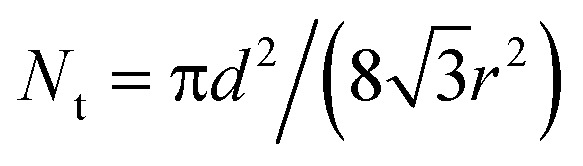
. Assuming that the water forms a contact angle of *θ* = 120° with the surface of the nanoparticles, the area of each particle that is wetted is *A*_1_ = 2π*r* ^2^(1 + cos *θ*) at zero applied pressure.

Considering a large water drop deposited on the surface, the wetted area per pillar is1
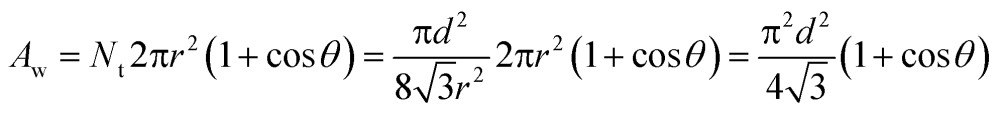



The total area (of the substrate and liquid–air interface) per pillar is

2 *A*_t_ = *P*^2^ – *N*_t_π*r*^2^sin^2^*θ* + *A*_w_.

Thus the wetted area fraction is3
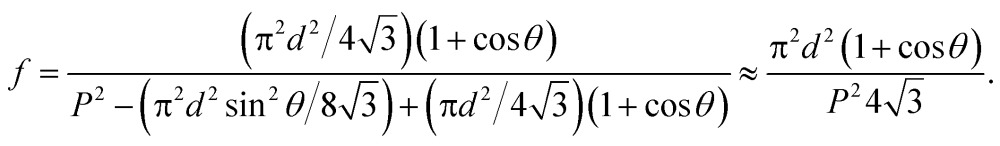



This fraction is independent of the size of particles.

## Results and discussion

**Fig. 2:**
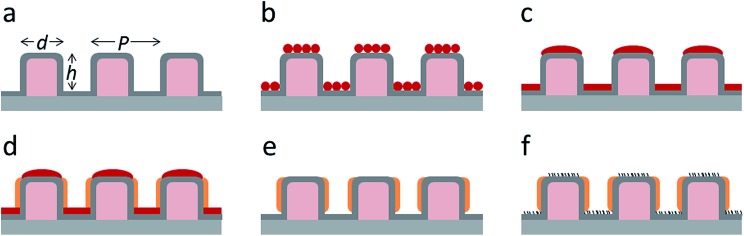
Concept: to fabricate functional Janus micropillar arrays, (a) the top faces of the micropillars were decorated with (b) one or more self-assembled monolayers of polymeric particles in a Langmuir trough. This covers and protects the top face after the particles were merged into a film by exposure to saturated toluene vapour (c), and the walls of the micropillars were chemically modified (d). After removing the protective polymer film (e) the top faces can be functionalized (f). The dimensions shown are not to scale.

**Fig. 3:**
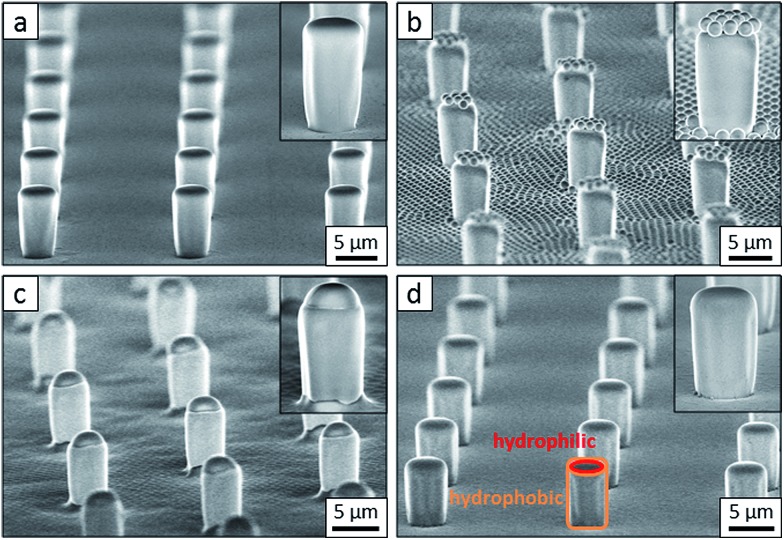
Scanning electron microscopy (SEM) images of a micropillar array after each step of the Janus micropillar fabrication technique. (a) Silica-coated SU-8 micropillars, (b) PS particle-decorated micropillars, (c) PS film-masked tops of micropillars and (d) Janus micropillars with hydrophilic silica tops (shown as a red-rimmed area) and hydrophobic sides (shown as an orange-rimmed area). The insets show a pillar at higher magnification. The dimensions of the micropillars are *d* = 4 μm, *P* = 20 μm, *h* = 9 μm, and *f* = 3%.

**Fig. 4:**
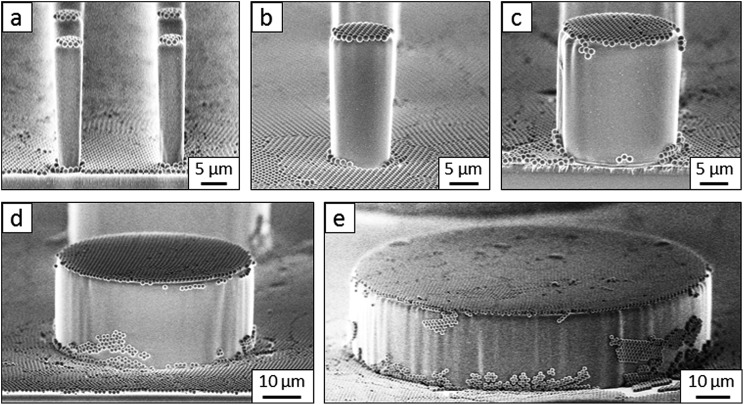
Scanning electron microscopy (SEM) images of particle-coated micropillars of varying diameters: 6 μm (a), 11 μm (b), 22 μm (c), 53 μm (d), and 105 μm (e). The height of the micropillars is *h* = 23 μm.

Flat-top cylindrical SU-8 micropillars with different diameters *d*, pitches *P*, heights *h*, and surface fractions were prepared on glass substrates using photolithography.^30^ After the epoxy-based photoresist surface was treated with an O_2_ plasma, a Stöber reaction^33,36^ was performed to coat the micropillars with an approximately 70 nm thick silica shell (Fig. 2a and 3a). The silica layer increased the mechanical stability of the micropillars. Some micropillar arrays were hydrophobised after coating with a silica shell (Fig. 2a). Most of the silica-coated micropillar arrays were decorated with a monolayer of hexagonally arranged polystyrene (PS) particles. These samples were either used to investigate the influence of the overhangs on the wetting properties (Fig. 2b, 3b, and 4) or to protect the top face during the modification of the sidewalls (Fig. 2c and 3c). To coat the top faces of micropillars with particles the substrates were put into a Langmuir trough.^37^ A droplet of the dispersion was deposited at the air–water interface to induce the formation of a self-assembled monolayer of particles. Then, the water level was lowered, and the micropillar tops and bottoms were homogeneously decorated. This method can be applied to decorate small as well as large micropillars with a well-defined monolayer of particles. Only occasionally, a few particles can be found at the sidewalls. This can happen if the monolayer was compressed slightly too fast or too much (Fig. 4). The particles at the bottom of the substrate will not affect the wetting behaviour as long as the drop stays in the Cassie state. One part of the particle pillars was hydrophobised and the remaining part was subsequently exposed to saturated toluene vapour. The adsorbed toluene softened the PS particles such that they formed a homogeneous film (Fig. 2c and 3c) that completely protected the micropillar tops while the side walls were hydrophobised with the semifluorinated silane 1*H*,1*H*,2*H*,2*H*-perfluorooctyldimethylchlorosilane to decrease the surface energy (Fig. 2d). The PS film was subsequently washed away, leaving the micropillars with hydrophobic sidewalls and hydrophilic top faces, which are known as Janus micropillars (Fig. 2e and 3d). The exposed silica surface of the micropillar tops could then be selectively modified either to precisely control the hydrophobic–hydrophilic characteristics or to enable the attachment of specific molecules (Fig. 2f).

We first investigated the wetting behaviour of fully fluorinated micropillars with a smooth top surface (Fig. 3a, termed “P”), fully fluorinated micropillars coated with a monolayer of particles (Fig. 3b and c termed “PP”) and micropillars with a hydrophilic silicon oxide top surface and hydrophobic walls (Fig. 3d, termed “JP”). The flat-top (P) and particle-coated (PP) micropillar arrays with low surface fractions, *f* = 5–6%, exhibited a roll-off angle, *α*, and hysteresis in the contact angle, Δ*θ* = *θ*_A_ – *θ*_R_, that was less than or equal to 10° (Table 1). The apparent advancing contact angle, *θ*_A_, was roughly the same for all surfaces, with a value of 155–159°. Low roll-off and high advancing and receding angles were better achieved with small structures. Micropillar arrays (P, PP) with higher surface fractions, *f* = 20–23%, exhibited significantly higher roll-off angles and contact angle hysteresis, where *α* attained values up to 32° and Δ*θ* attained values up to 27° (Table 2). However, the advancing angle remained always well above 150°.

**Table 1:** Comparison of the wetting behavior of flat-top micropillar (P) and particle-coated micropillar (PP) arrays with low surface fraction *f* and of varying dimensions (*i.e.*, diameter *d* and pitch *P*). Listed are the apparent advancing and receding contact angles, *θ*_A_ and *θ*_R_, the hysteresis, Δ*θ*, and the lateral and diagonal roll-off angles, *α* and *α*_D_. The height of the micropillars is *h* = 23 μm. The standard deviation was calculated from five independent measurements, each. The surface fraction of the particle-decorated micropillars was calculated according to eqn (3). For comparison we measured the apparent contact angles of an equally treated flat SU8 surface after coating with a silica shell and hydrophobization; *θ*_A_ (flat) = 124° ± 2°, and *θ*_R_ (flat) = 85° ± 5°

	*d*/μm	*P*/μm	*f*%	*θ*_A_	*θ*_R_	Δ*θ*	*α*	*α*_D_
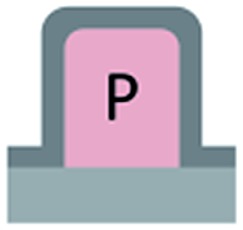	6	21	6	156° ± 2°	149° ± 1°	7°	6° ± 1°	5° ± 1°
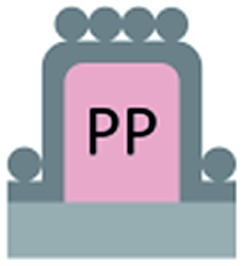	6	21	6	157° ± 2°	152° ± 1°	5°	4° ± 1°	4° ± 1°
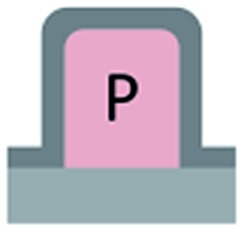	11	41	6	157° ± 2°	148° ± 1°	9°	6° ± 1°	6° ± 1°
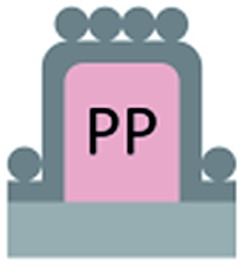	11	41	5	156° ± 2°	150° ± 1°	6°	6° ± 1°	4° ± 1°
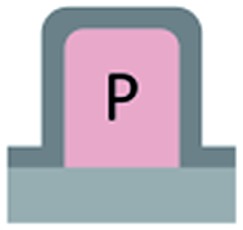	26	102	5	157° ± 2°	147° ± 2°	10°	9° ± 1°	9° ± 2°
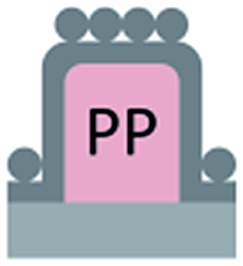	26	102	5	156° ± 2°	147° ± 1°	9°	8° ± 1°	7° ± 2°
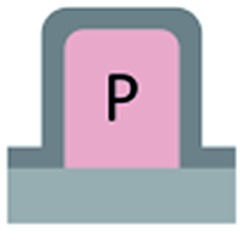	54	208	5	155° ± 2°	145° ± 1°	10°	11° ± 2°	10° ± 2°
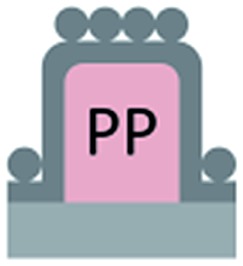	54	208	5	157° ± 2°	148° ± 2°	9°	8° ± 2°	8° ± 1°

**Table 2:** Comparison of the wetting behavior of flat-top micropillar (P) and particle-coated micropillar (PP) arrays with higher surface fraction *f* and of varying dimensions (*i.e.*, diameter *d* and pitch *P*). Listed are the apparent advancing and receding contact angles, *θ*_A_ and *θ*_R_, the hysteresis, Δ*θ*, and the lateral and diagonal roll-off angles, *α* and *α*_D_. The height of the micropillars is *h* = 23 μm. The standard deviation was calculated from five independent measurements, each. The surface fraction of the particle-decorated micropillars was calculated according to eqn (3)

	*d*/μm	*P*/μm	*f*%	*θ*_A_	*θ*_R_	Δ*θ*	*α*	*α*_D_
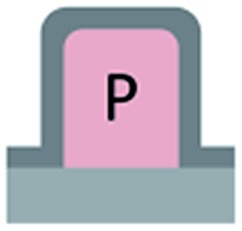	11	21	22	157° ± 2°	139° ± 1°	18°	20° ± 2°	19° ± 1°
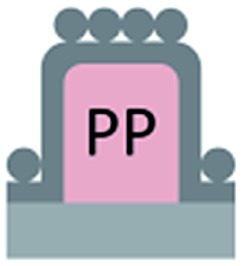	11	21	20	157° ± 2°	142° ± 1°	15°	15° ± 1°	14° ± 2°
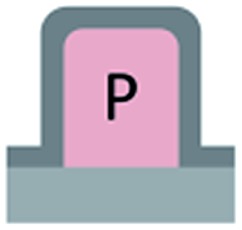	22	41	23	156° ± 2°	139° ± 1°	17°	21° ± 2°	20° ± 1°
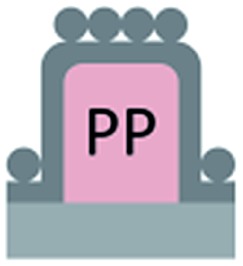	22	41	21	157° ± 2°	142° ± 1°	15°	17° ± 1°	14° ± 1°
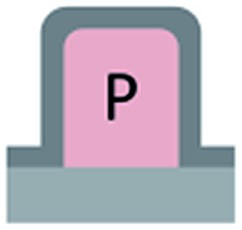	53	106	20	156° ± 2°	137° ± 2°	19°	23° ± 2°	21° ± 3°
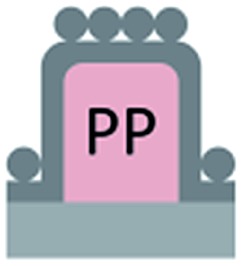	53	106	18	157° ± 3°	139° ± 2°	18°	21° ± 1°	18° ± 1°
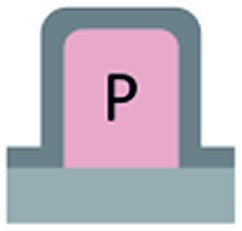	105	207	20	155° ± 2°	128° ±2°	27°	32° ± 2°	29° ± 4°
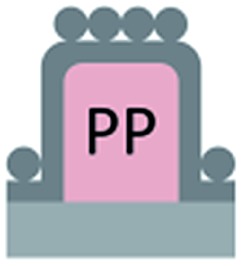	105	207	18	155° ± 2°	135° ± 1°	20°	24° ± 1°	19° ± 1°

**Fig. 5:**
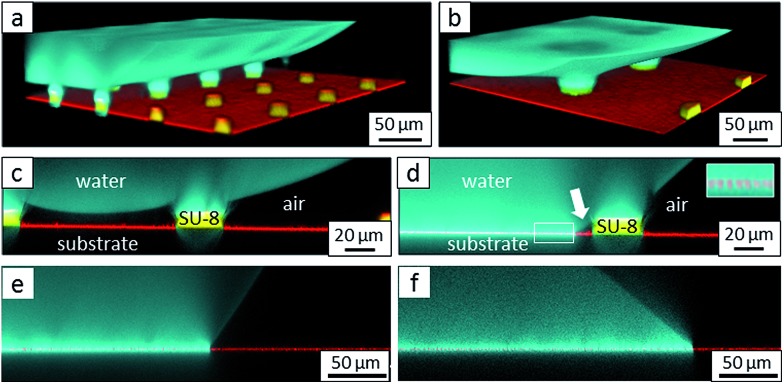
Scanning electron microscopy images of micropillars and confocal microscopy images of sessile water drops. (a and b): 3D laser scanning confocal microscopy images of a sessile water drop (cyan) on particle micropillars (yellow) with (a) *d* = 26 μm and *P* = 102 μm and (b) *d* = 54 μm and *P* = 208 μm; both water and SU-8 micropillars were dyed, where the emission wavelengths of the dyes were well separated to enable simultaneous detection; the water-soluble perylene-diimide-based dye (WS-PDI) was not interfacially active,^43^ and the light reflected at the substrate–air interface (red) was simultaneously recorded. The 3D images were obtained by superposing the fluorescence and reflection images; (c and d): vertical section through a water drop resting on an array of particle micropillars (*d* = 54 μm and *P* = 208 μm) (c) in the micro- and nano-Cassie states and (d) in the micro-Wenzel and nano-Cassie states. A small air bubble remained at the side of the pillar (d, white arrow); the inset in d (white square) illustrates that the “nano-air pockets” (red spots) were stable even in the micro-Wenzel state; red: reflected light at the air–glass and air–SU-8 interface; white: reflections at the glass-water interface; the refractive indices are as follows: *n*_air_ = 1.0, *n*_water_ = 1.33, *n*_glass_ = 1.46, and *n*_SU-8_ = 1.6.

To verify that despite the large roll-off angles the drops were still separated from the substrate by an air cushion, we imaged the drops using laser scanning confocal microscopy (LSCM).^30^ For this purpose, we labelled the SU-8 micropillars with a hydrophobic perylene-monoimide-based fluorophore (PMI).^31^ A water soluble perylene-diimide-based dye (WS-PDI) was added to the water phase. We simultaneously recorded the light reflected from the interfaces. The superposition of the fluorescent (cyan and yellow) and reflection (red) images showed the morphology of the water–air and micropillar–air interfaces with a horizontal resolution of ≈250 nm and a vertical resolution of ≈1 μm. Indeed, the air cushion (black) separating the drop (cyan) and the substrate was clearly visible (Fig. 5a–c). The same result was found for the fully fluorinated micropillar arrays and the micropillar arrays with hydrophilic top surfaces (Fig. 8b). Notably, all drops were well separated from the substrate by an air cushion, proving that they remained in the Cassie state (Tables 1 and 2). The “Cassie state” refers to a configuration in which the droplet is separated from the substrate by an air cushion but the roll-off angle can exceed 10°.^38,39^ This verifies that the Cassie state does not necessarily correspond to superhydrophobic behaviour.

The additional particle layer on top of the micropillars reduced the roll-off angle and the contact angle hysteresis (Tables 1 and 2). We attribute this result to the overhangs formed by the spheres (Fig. 3b, inset), which should enhance superhydrophobicity.^40,41^ The overhangs produced a so-called “nano-Cassie state”^42^ in which air was trapped between the micropillars (“micro-Cassie state”) and in the interstitials of the particles. This nano-air layer could be imaged by recording the reflection of light from the water–air interface (red line and spots in Fig. 5d–f) To test whether the nano-air layer is also stable for a pure particle layer on a glass substrate we measured the reflected light for an advancing and a receding drop; *θ*_A_ (monolayer) = 125° ± 1°, *θ*_R_ (monolayer) = 39° ± 3°. In both cases the nano-air layer remained stable (Fig. 5e and f). For the particle coated micropillar arrays a hierarchy of Cassie states was observed. The nano-Cassie-state remained stable even after the drop was forced into the “micro-Wenzel state” (Fig. 5d). The transition to the micro-Wenzel state was caused by an evaporation-induced increase in the Laplace pressure. The particle layer on top of the pillars locally induced a nano-Cassie state which reduced line pinning. This resulted in the increase in the receding contact angle, *θ*_R_, of up to 7° and a decrease in the roll-off angle, *α*, of up to 10° (Tables 1 and 2).

**Fig. 6:**
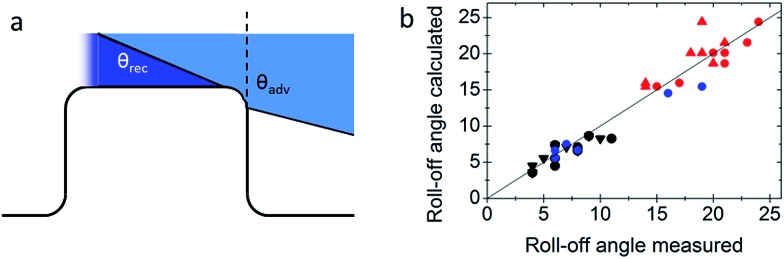
Roll-off angles: (a) schematic of the water deposition on a micropillar, (b) comparison of experimentally determined roll-off angles and roll-off angles calculated using the El-Sherbini equation; black triangles: P, *f* = 5–6%; black spheres: PP, *f* = 20–23%; blue spheres: P, *f* = 3–6%, red triangles: JPP, *f* = 3–6%; and red spheres: JP, *f* = 3–6% (Tables 1–3); the black symbols indicate superhydrophobic surfaces, and the red symbols indicate surfaces in the Cassie state.

The hydrophilic tops of the micropillars did not significantly affect the advancing contact angle, whereas the receding angle varied. The apparent advancing contact angle did not depend on the surface chemistry of the top faces of the micropillars. This result demonstrates that the apparent advancing contact angle was determined by contact line pinning at the sides of the micropillars, in accordance with the Gibbs criterion^44^ (Fig. 6a). In contrast, the apparent receding contact angle depended on the surface chemistry and the shape of the three-phase contact line of the micropillars. On the receding side, the three-phase contact line had to slide over the top face of a micropillar, which was hindered on a hydrophilic surface relative to a hydrophobic surface. Therefore, the contact angle hysteresis increased with the area fraction and the hydrophilicity of the micropillars.

To further support the hypothesis that the force per unit line rather than thermodynamics determined the macroscopic wetting behaviour, we related the contact angle hysteresis to the observed roll-off angles. The roll-off angle was obtained by balancing the surface tension force around the periphery of the drop with the gravitational force, *ρVg*sin *α*. Here, *ρ* is the density of the liquid, *g* = 9.81 m s^–2^, and *V* is the volume of the drop. This force balance yields *ρVg*sin *α* = *kγw*(cos *θ*_R_ – cos *θ*_A_),^30^ where *w* is the width of the apparent contact area. The width for such high contact angles is difficult to observe in a sliding experiment. Therefore, we calculated the width from the drop volume and the receding contact angle. For small drops that have a spherical cap shape, geometrical considerations yield *w* = (24*V*/π*β*)^1/3^sin *θ* with *β* = (1 – cos *θ*)^2^(2 + cos *θ*).^45^ The constant *k* depends on the precise shape of the drop just before it begins to slide. The values of *k* between 4/π and 2.0 have been reported for flat surfaces.^45,46^ The observed value of *k* = 2.0 (Fig. 6b) indicates that each individual micropillar exhibited strong contact line hysteresis,^47^ probably because of the formation of liquid microbridges.^30,48^ The hysteresis was only low at a macroscopic scale.

**Fig. 7:**
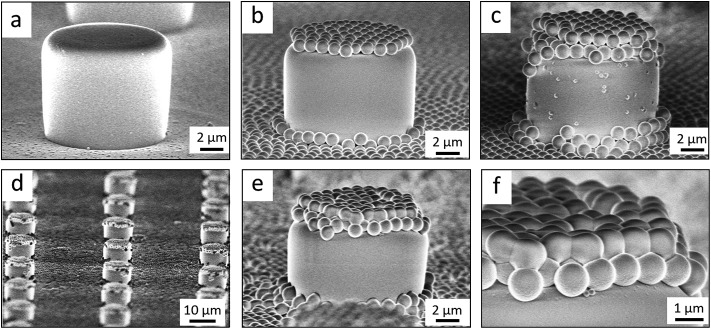
SEM images of silica-coated SU-8 micropillars. (a) Silica-coated SU8 micropillar, (b) particle micropillar, (c–f) particle micropillars that are decorated with an additional layer of PS particles before (c) and after (d–f) exposure to toluene vapour (dimensions: *d* = 11 μm, *P* = 40 μm, and *h* = 9 μm).

So far, we have shown two functions of the particle layer: on the one hand, polymeric particles can be merged together to shield the top faces of the pillars while hydrophobizing the walls. On the other hand the particles induce overhangs and thereby increase the stability of the Cassie state. Aiming to combine both functions, we proceeded as follows: first, a monolayer of PS particles was deposited onto the micropillar arrays. Next, the particle decorated micropillars were coated with a silica shell (Fig. 7a and b). This prevents swelling of the PS particles during further treatment. Then, a second monolayer of PS particles was deposited on top of the micropillars (Fig. 7c). The pillar arrays, now decorated with two layers of particles, were exposed to toluene vapour. This induced film formation of the topmost particle layer while the underlying layer could not swell due to its coating with a silica shell (Fig. 7d–f). After the sidewalls were hydrophobised the PS film was removed by thorough washing with different solvents resulting in Janus particle-covered micropillars with silica top faces. The increased stability of the Cassie state was reflected in the decreased roll-off angle and the increased contact angles (Tables 1 and 2). To increase the robustness of the Cassie state, it was important that the hydrophilic domain did not extend beyond the rim of the micropillar but was well restricted to its top face.

**Fig. 8:**
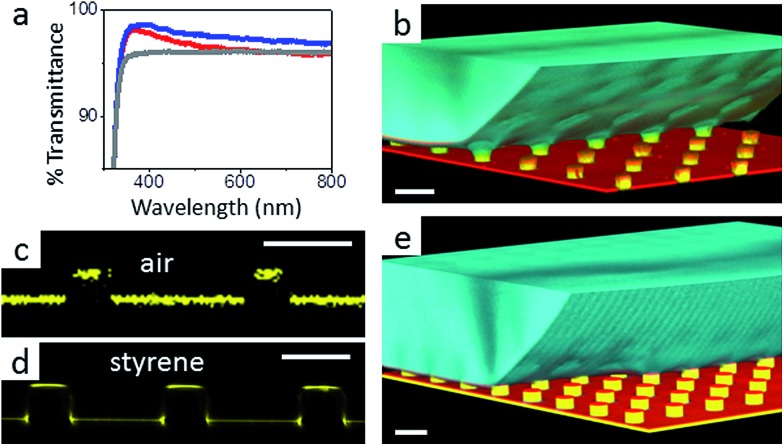
Functionalized micropillar arrays. (a) Transmittance spectra of Janus micropillars (blue line: Janus micropillars with *d* = 4 μm, red line: Janus micropillars with *d* = 11 μm and grey line: bare glass substrate). Enhanced light transmission, particularly at short wavelengths, was caused by reduced reflectivity;^49^ (b) 3D laser scanning confocal microscopy images showing a sessile water drop deposited onto Janus micropillars (*d* = 11 μm, *P* = 40 μm, and *h* = 9 μm); the silica micropillar tops were selectively functionalised with (c) the fluorescently labelled peptide, GALA, and (d and e) fluorescein-5-isocyanate, as verified by LSCM in fluorescence mode (c and d); the FITC-modified micropillars were wetted with styrene to minimise reflections at the substrate surface. (e) The functionalised substrates remained in the Cassie state; white scale bars: 20 μm.

To demonstrate that the silica top faces can be chemically functionalized by hydrophilic molecules (Fig. 2f), we functionalised the tops of the micropillars with the fluorophore fluorescein-5-isothiocyanate (FITC) and a fluorescently labelled 30-amino-acid-long synthetic peptide with a glutamic acid–alanine–leucine–alanine (GALA) repeat that has been developed for drug and gene delivery.^50,51^ In both cases, the silica top surface was first functionalised with aminopropyl-triethoxysilane. FITC was directly bound to the amine groups.^34^ The GALA repeat was attached using a strategy based on azide–alkyne click chemistry. The amine groups were first coupled to the active ester dibenzylcyclooctyne-*N*-hydroxysuccinimide (DBCO-NHS ester). Then, the fluorescently labelled GALA, equipped with an azide group, was introduced during the synthesis using azido-ε-lysine and was bound to the DBCO-modified surface. UV-Vis spectroscopy of the Janus micropillar arrays on glass slides revealed excellent transparency that exceeded even that of a bare glass substrate (Fig. 8a). The hydrophilisation of the top faces did not change the advancing contact angle within experimental accuracy (Table 3). However, the receding contact angle slightly decreased and the roll-off angle increased, hinting towards increased adhesion. The 3D laser scanning confocal microscopy images demonstrated that a sessile water drop deposited onto an array of Janus micropillars remained in the Cassie state (Fig. 8b). The fluorescence images indicate that no GALA (Fig. 8c) or FITC molecules (Fig. 8d) were attached to the micropillar sidewalls. In contrast, strong fluorescence was observed at the top faces of the micropillars and at the bottom of the substrate. To ensure that the detected light was not based on reflections at the solid–air interface, we wetted the FITC-modified Janus micropillars with styrene. The refractive indices of styrene (*n*_styrene_ = 1.55) and SU-8 (*n*_SU-8_ = 1.6) are well matched. Hence, the detected light originated solely from the emissions of the attached FITC molecules (Fig. 8d). The deposited water drop remained in the Cassie state proving the robustness of the Cassie state after chemical modification of the top faces (Fig. 8e).

**Table 3:** Wetting properties of pillar (P), Janus pillar (JP), particle pillar (PP) and Janus particle pillar (JPP) arrays of different dimensions (*h* = 9 μm)

Sample	*d*/μm	*P*/μm	*f*%	*θ*_ST_	*θ*_A_	*θ*_R_	Δ*θ*	*α*
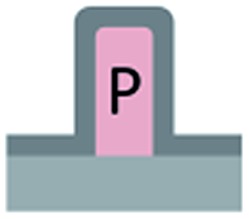	4	20	3	155° ± 1°	159° ± 1°	151° ± 2°	8°	6° ± 2°
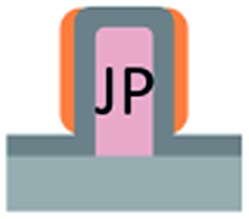	4	20	3	153° ± 1°	156° ± 1°	148° ± 1°	8°	8° ± 1°
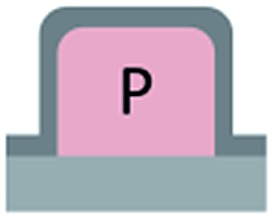	11	40	6	154° ± 1°	159° ± 1°	149° ± 1°	10°	7° ± 1°
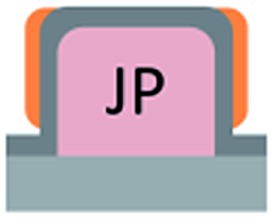	11	40	6	150° ± 1°	156° ± 1°	141° ± 1°	15°	19° ± 1°
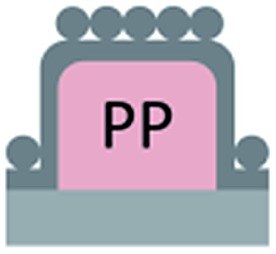	11	40	1	155° ± 1°	158° ± 1°	149° ± 1°	9°	6° ± 1°
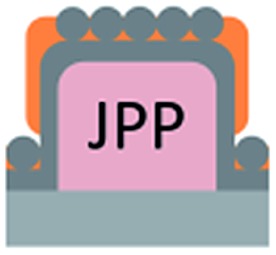	11	40	—	151° ± 1°	157° ± 1°	142° ± 2°	15°	16° ± 2°

## Conclusions and comments

The apparent advancing contact angle of water and the stability of the Cassie state are determined by the walls of the micropillars, and not by the top faces. The apparent receding contact angle decreases when the top faces are hydrophilised, due to increased pinning of the receding contact line. To increase the receding contact angle, mono- and bilayers of particles were deposited on top of the micropillars. These particles induce a nano-Cassie state and facilitate the sliding of the receding edge of a water drop by breaking the contact line.

This decoupling of the apparent advancing and receding contact angles enables the fabrication of macroscopically superhydrophobic arrays of micropillars with locally hydrophilic silica top surfaces. The silica top surface of the micropillars allows for facile and versatile functionalisation by a variety of different coupling chemistries. The results offer new perspectives in surface-tension-confined microfluidics^2,10,12,52^ cell–water condensation,^53^ slip reduction,^54,55^ or drop impact.^56,57^ Here, we designed superhydrophobic microarrays up to 250 000 hydrophilic spots per square centimetre. The diameters of the hydrophilic spots of the Janus micropillars are in the size range of the cell diameters; therefore, the developed strategy has potential applications, *e.g.*, a cell or single bacteria could be attached and immobilised on the top of each micropillar to investigate cell–cell communication, cell growth and proliferation.^58^ Notably, the observation that hydrophilic top surfaces do not affect the robustness of the Cassie state implies that the Cassie state corresponds to remarkable damage tolerance. This result explains why superhydrophobic surfaces that are exposed to strong friction still can exhibit high contact angles.^59^
